# Metabolic and Redox Biomarkers in Skeletal Muscle Underlie Physiological Adaptations of Two Estivating Anuran Species in a South American Semi-arid Environment

**DOI:** 10.3389/fphys.2021.769833

**Published:** 2021-12-09

**Authors:** Daniel C. Moreira, Juan M. Carvajalino-Fernández, Carlos A. Navas, José E. de Carvalho, Marcelo Hermes-Lima

**Affiliations:** ^1^Núcleo de Pesquisa em Morfologia e Imunologia Aplicada, Área de Morfologia, Faculdade de Medicina, Universidade de Brasília, Brasília, Brazil; ^2^Departamento de Biologia Celular, Instituto de Ciências Biológicas, Universidade de Brasília, Brasília, Brazil; ^3^Laboratory of Adaptations to Extreme Environments and Global Change Biology, Universidad Colegio Mayor de Cundinamarca, Bogotá, Colombia; ^4^Departamento de Fisiologia, Biosciences Institute, Universidade de São Paulo, São Paulo, Brazil; ^5^Departamento de Ecologia e Biologia Evolutiva, Universidade Federal de São Paulo, Diadema, Brazil

**Keywords:** Caatinga, glutathione, preparation for oxidative stress, reactive oxygen species, amphibia, antioxidant, *Pleurodema diplolister*, *Proceratophrys cristiceps*

## Abstract

The upregulation of endogenous antioxidants (i.e., preparation for oxidative stress, POS) is part of the biochemical responses underlying the adaptation of animals to adverse environments. Despite the phylogenetic diversity of animals in which POS has been described, most studies focus on animals under controlled laboratory conditions. To address this limitation, we have recently assessed the redox metabolism in the skeletal muscle of *Proceratophrys cristiceps* estivating under natural settings in the Caatinga. Here, we analyzed biochemical biomarkers in the muscle of another Caatinga species, *Pleurodema diplolister*, during the rainy (active) and dry (estivating frogs) seasons. We aimed to determine whether *P. diplolister* enhances its antioxidants during estivation under field conditions and to identify any effect of species on the biochemical responses of *P. diplolister* and *P. cristiceps* associated with estivation. To do so, we measured the activities of representative enzymes of intermediary metabolism and antioxidant systems, as well as glutathione and protein carbonyl levels, in the skeletal muscle of *P. diplolister*. Our findings revealed the suppression of oxidative metabolism and activation of antioxidant enzymes in estivating *P. diplolister* compared with active specimens. No changes in oxidative damage to proteins were observed and estivating *P. diplolister* had lower levels of disulfide glutathione (GSSG) and disulfide-to-total glutathione ratio (GSSG/tGSH) than those observed in active individuals. When data for *P. diplolister* and *P. cristiceps* were assembled and analyzed, significant effects of species were detected on the activities of metabolic enzymes (citrate synthase, isocitric dehydrogenase, malic enzyme, and creatine kinase) and antioxidant enzymes (catalase, glutathione peroxidase and glutathione transferase), as well as on GSSG/tGSH ratio. Such effects might underlie the physiological and behavioral differences between these two species that share the same microhabitat and survival strategy (i.e., to estivate) during the dry season. Despite some peculiarities, which reflect the physiological diversity of the mechanisms associated with estivation in the Brazilian Caatinga, both *P. diplolister* and *P. cristiceps* seem to balance the suppression of oxidative pathways, the maintenance of the capacity of oxygen-independent pathways, and the activation of endogenous antioxidants to preserve muscle function and be ready to resume activity whenever the unpredictable rainy period arrives.

## Introduction

Many animal species rely on their ability to suppress global metabolism and survive under harsh ambient conditions until the environment becomes suitable for growth, development, and reproduction again (Withers and Cooper, 2010; Hahn and Denlinger, 2011; Storey and Storey, 2012; Ruf and Geiser, 2015). Such ability is fundamental to survive during long periods of limited environmental resources, such as winters or dry seasons, when animals minimize their needs for resources and function “in the slow lane” (Storey, 2002). Many molecular players that drive the reorganization of metabolism, reprioritization of specific pathways, and cell preservation during metabolic depression have been unveiled (Storey and Storey, 2011, 2012; Storey and Wu, 2013; Storey, 2015; Biggar and Storey, 2018; Hadj-Moussa et al., 2018). Among them, a large body of research indicates that a common response of animals challenged by adverse environments is the upregulation of endogenous antioxidants (Hermes-Lima and Zenteno-Savín, 2002; Welker et al., 2013; Moreira et al., 2016), a phenomenon called preparation for oxidative stress (POS; Hermes-Lima et al., 1998, 2015; Giraud-Billoud et al., 2019). Given the large number of studies on the modulation of antioxidants conducted with animals under artificially controlled settings, we have previously set the challenge of assessing POS under natural conditions (Moreira et al., 2017). This challenge was met by few recent studies (Ondei et al., 2020; Moreira et al., 2021; Patnaik and Sahoo, 2021), including one that evaluated the modulation of endogenous antioxidants in the muscle of *Proceratophrys cristiceps* (Anura: Odontophrynidae) from the Brazilian Caatinga (Moreira et al., 2020). In addition to the scarcity of studies under field conditions, it is still not known if POS can be generalized to other anuran species estivating in semi-arid environments.

The Caatinga is a semi-arid biome that occupies approximately 10% of the Brazilian territory within the latitude range of 2°54′–17°21′S (Prado, 2003). This morphoclimatic domain is characterized by high incidence of solar radiation, high average annual temperature, low relative humidity, and seasonal, yet irregular rainfall regimes (Prado, 2003). The rainy season is limited to a period of a few months, usually starting in January. The pattern of precipitation, however, is widely variable, so the duration of the drought is uncertain from year to year. In the Caatinga, some regions remain without any rain for entire years (Nimer, 1972). Still, the number of amphibian species that inhabit this extreme biome is estimated to be 73–120 (Camardelli and Napoli, 2012; Toledo and Batista, 2012). Anurans adapted to the Caatinga show morphological, behavioral, and physiological adaptations to maintain viability during long periods of drought (Navas et al., 2004; Jared et al., 2019). Like some Australian (e.g., *Neobatrachus* and *Cyclorana*; Withers, 1993; Kayes et al., 2009) and North American (e.g., *Scaphiopus couchii*; McClanahan, 1967) species, some Caatinga frogs burrow and reduce their aerobic metabolic rate during the dry season, using estivation as a survival strategy (Carvalho et al., 2010). *Pleurodema diplolister* is one of the estivating species of the Caatinga whose biology has been considerably studied.

Previous research on the ecophysiology of *P. diplolister* has found that its reproductive physiology and immunity are markedly affected by the seasonality of the Caatinga (Madelaire and Gomes, 2016; Madelaire et al., 2017). Circulating levels of androgens, corticosterone, and leukocytes, as well as the inflammatory response to immunological challenges, are suppressed during the dry season (Madelaire and Gomes, 2016; Madelaire et al., 2017). More recently, adjustments in the regulation of energy turnover, protein synthesis, and proteostasis in the skeletal muscle of *P. diplolister* during estivation have also been described (Madelaire et al., 2020). In brief, key biomarkers indicate the maintenance of aerobic capacity, suppression of protein synthesis, and activation of cell survival processes in the muscle of *P. diplolister* during the dry season (Madelaire et al., 2020). These findings were interpreted as a strategy to limit the loss of musculoskeletal performance despite the long period of inactivity and lack of environmental resources (Madelaire et al., 2020). Indeed, estivating anurans are extraordinarily resistant to muscle atrophy (Hudson and Franklin, 2002; Hudson et al., 2006; Reilly and Franklin, 2016), whose underlying mechanism has a strong contribution of reactive oxygen species and antioxidant systems (Powers et al., 2011; Talbert et al., 2013). Still, the occurrence of any adjustment of antioxidant systems in the skeletal muscle of *P. diplolister* remains unexplored.

We have previously found that another estivating frog, *P. cristiceps*, upregulate its endogenous antioxidants during the dry season in the Brazilian Caatinga (i.e., estivating period; Moreira et al., 2020). Although *P. cristiceps* and *P. diplolister* present the same survival strategy (estivation) and share the same microhabitat during the dry season (Jared et al., 2019), they differ in some aspects. In addition to their phylogenetic distance, adult males of *P. cristiceps* are larger (~2.6-fold heavier) than those of *P. diplolister*, which exhibits higher resting aerobic metabolic rate (~3.8-fold faster, mass normalized) than *P. cristiceps* when assessed at the same season (Pereira, 2016). The species also differ in their responsiveness to external stimuli during the dry season. Estivating *P. diplolister* readily resumes locomotor activity in an escape response when disturbed (Pereira, 2016; Jared et al., 2019), whereas *P. cristiceps* remains rather inactive and takes minutes for its first apparent response (Pereira, 2016). The biochemical basis, if any, underlying such differences between these species are still unknown.

The objectives of this research were: (i) to determine whether *P. diplolister* enhance its muscular antioxidants during estivation under field conditions – i.e., does *P. diplolister* present the POS mechanism in its natural environment?; (ii) to investigate differences, if any, between the biochemical adjustments in the skeletal muscle of *P. diplolister* and *P. cristiceps*; and (iii) to identify metabolic differences between the two frog species that might explain their peculiarities in behavior and physiology. To do so, we measured the activities of representative enzymes involved in intermediary metabolism and antioxidant systems, as well as glutathione and protein carbonyl levels, in the skeletal muscle of active and estivating *P. diplolister*, collected during the rainy and dry seasons, respectively. Lastly, we also put our findings together with those of a similar previous study on antioxidants in the muscle of *P. cristiceps* collected in the same area (Moreira et al., 2020) to analyze the effect of species (i.e., *P. diplolister* or *P. cristiceps*) and metabolic state (i.e., active or estivating) on biochemical variables in these two species adapted to endure extreme and seasonal changes of water availability in the Brazilian Caatinga.

## Materials and Methods

### Animals

**Figure 1:**
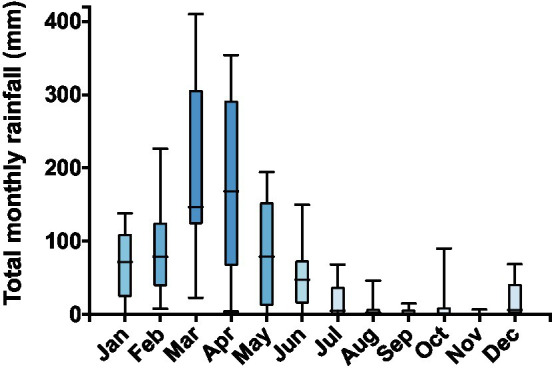
Total rainfall (mm) for each month from 2005 to 2012 as recorded in the Florânia meteorological station (6°6′36ʺS and 36°48′36ʺW) of the National Meteorological Institute (INMET), the closest station to the collection site. The Caatinga is characterized by a rainy season within the first 3–4months of the year and high annual variability of total precipitation levels. Data are shown as boxes (from the 25th to the 75th percentiles) and whiskers (from the lowest to the highest value) with the line inside the box marking the median.

Animals were collected inside a private area, Fazenda São Miguel (5°41′27″S, 36°26′19″W), near the municipality of Angicos, State of Rio Grande do Norte, Brazil. The farm has been inactive for a long time and is situated inside the domain of the Caatinga, a biome restricted to Northeastern Brazil. High average temperatures and prominent rainy seasons (usually in January–April) followed by dry seasons characterize this biome (Figure 1). Other peculiarity of the Caatinga is the unpredictability of the rain regime, which is evidenced by its highly variable monthly rainfall levels (Figure 1). During the dry season, some anuran species, such as *Rhinella granulosa* and *R. jimi*, remain active foraging in humid microhabitats (Madelaire et al., 2017), whereas other species, such as *Pleurodema diplolister*, *Proceratophrys cristiceps*, and *Physalaemus* spp., estivate buried under dry river beds until the arrival of the rainy season (Carvalho et al., 2010; Jared et al., 2019). Detailed information about the collection site, as well as the behavior and morphology of the estivating species, is available in a recent publication (Jared et al., 2019).

Adult specimens of *P. diplolister* and *P. cristiceps* were collected during four expeditions (2005–2012). Active animals were collected on the surface of the sandy soil during the rainy season. Estivating animals were collected during the dry season after excavating dry river beds and locating the animals buried in the soil. Once located and identified, whole animals were immediately frozen in liquid nitrogen to avoid any manipulation effects, and kept at −80°C until biochemical analyses. Active *P. diplolister* were collected in December 2005 and estivating *P. diplolister* were collected in August 2012. Procedures were approved by Universidade de São Paulo Animal Care and Use Committee (168/2012), under permits from IBAMA (02010.003380/04-82 and 14836-1) and SisGen (A0F0B20).

### Meteorological Data

Monthly rainfall totals for 2005–2012 were obtained from a station of the National Meteorological Institute (INMET) located at 6°6′36″S and 36°48′36″W, the closest station to the collection site.

### Enzyme Activity

To measure the activity of metabolic and antioxidant enzymes, the whole muscle mass surrounding the right femur of each animal was homogenized as previously described (Moreira et al., 2020). Briefly, the muscle tissue was mechanically homogenized in 50mM Tris (pH 7.4), 1mM EDTA, 1mM phenylmethylsulfonyl fluoride and 1:500 (*v*/*v*) protease inhibitor cocktail (P8340, Sigma-Aldrich, St. Louis, MO, United States). Then, the homogenate was centrifuged at 10,000×*g* for 15min at 4°C. The supernatants were transferred to new tubes, kept on ice and used for the determination of enzymatic activity in the same day of the homogenization.

The activities of citrate synthase (CS), isocitric dehydrogenase (ICDH), malic enzyme (ME), pyruvate kinase (PK), lactic dehydrogenase (LDH), creatine kinase (CK), glutathione transferase (GST), total superoxide dismutase (tSOD), catalase, glutathione peroxidase using H_2_O_2_ as substrate (GPX), and glutathione peroxidase using cumene hydroperoxide as substrate (tGPX) were measured using kinetic spectrophotometric assays as previous described (Moreira et al., 2020 and references therein).

Enzymatic activity was normalized to the concentration of protein in the supernatants, which was measured using coomassie brilliant blue G-250 (Bradford, 1976), as previously described (Moreira et al., 2020).

### Glutathione and Protein Oxidation

To measure the levels of glutathione (reduced and disulfide forms) and protein carbonyl groups, the whole muscle mass surrounding the left femur of each animal was homogenized as previously described (Moreira et al., 2020). Briefly, the muscle tissue was mechanically homogenized in 10% (*w*/*v*) trichloroacetic acid (TCA), aliquoted in three tubes, and centrifuged for 10,000×*g* for 10min at 4°C. The supernatants were assessed immediately to measure levels of total glutathione (tGSH) and disulfide glutathione (GSSG) using the enzymatic recycling method (Rahman et al., 2006), as previous described (Moreira et al., 2020). Reduced glutathione (GSH) levels were calculated using the formula: GSH=tGSH−2×GSSG. Two pellets were stored at −80°C until the determination of protein carbonyl concentration using 2,4-dinitrophenylhydrazine in a colorimetric assay (Fields and Dixon, 1971), as previously described (Moreira et al., 2020).

The third pellet was used to measure protein levels and normalize the concentrations of glutathione and protein carbonyl groups. Protein concentration in the pellets was determined using coomassie brilliant blue G-250 (Bradford, 1976) after resuspending the pellets in 100mM NaOH, as previously described (Moreira et al., 2020).

### Statistics

First, we analyzed the biochemical data for *P. diplolister* as previously (Moreira et al., 2020). Briefly, data had their distribution tested using the Shapiro–Wilk normality test. We used unpaired *t* tests (two-tailed) for data with a normal distribution and Mann–Whitney tests (two-tailed) for data with a distribution other than normal to compare active with estivating *P. diplolister*. Then, we compiled the original data generated in the present study with those already published in a similar study (Moreira et al., 2020) and conducted a two-way ANOVA to analyze the effects of species and metabolic state on each biochemical variable. Finally, we also analyzed the compiled data using a principal component analysis (PCA) in Minitab 19 (Minitab, State College, PA, United States). Considering the strong (*R*^2^=0.9960) and significant (Pearson correlation, *p*<0.0001) correlation between tGSH and GSH levels among all animals, we excluded tGSH levels in the PCA to avoid distortions of the outcome; all other parameters were included. Normality tests, *t* tests, Mann–Whitney tests, Pearson correlation and two-way ANOVA tests were conducted using GraphPad Prism 8 (GraphPad, San Diego, CA, United States). Graphs were created using GraphPad Prism 8. For all analyses values of *p* below 0.05 were considered to indicate statistically significant differences/effects.

## Results

### Estivating vs. Active *Pleurodema diplolister*

**Figure 2:**
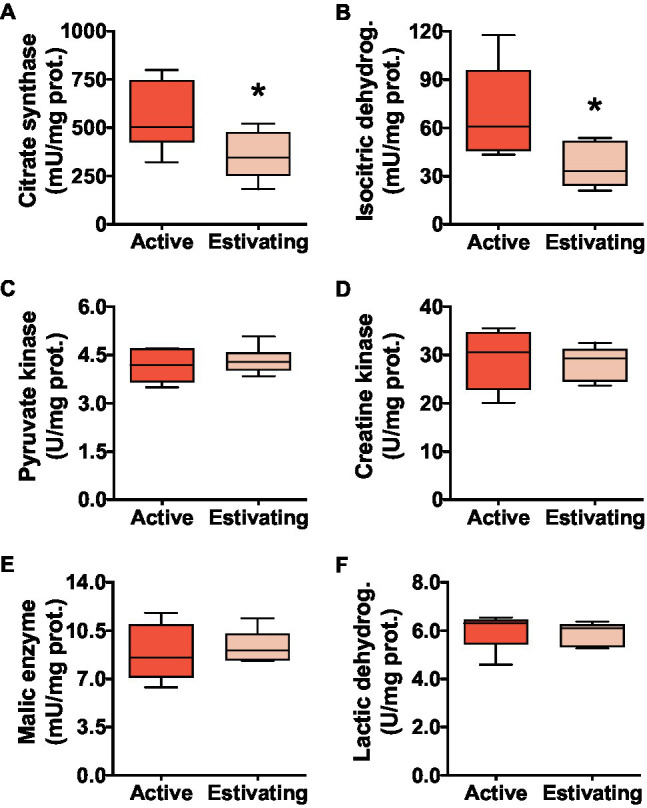
Activities of enzymes that catalyze key steps in metabolic pathways in the skeletal muscle of *Pleurodema diplolister* collected during the dry season (estivating) or the rainy season (active). **(A)** Citrate synthase. **(B)** Isocitric dehydrogenase. **(C)** Pyruvate kinase. **(D)** Creatine kinase. **(E)** Malic enzyme. **(F)** Lactic dehydrogenase. The asterisk (*) denotes significant differences between active and estivating groups. *N*=6.

The activities of CS and ICDH, enzymes of oxidative metabolism, were lower in the muscle of estivating frogs than those in active frogs. Compared with active animals, CS activity was 35.8% lower (Figure 2A) and ICDH activity was 47.9% lower (Figure 2B) in the muscle of estivating *P. diplolister*. The activity of muscular pyruvate kinase (PK), which catalyzes a key step in glycolysis, did not differ between active and estivating *P. diplolister* (Figure 2C). The activities of enzymes involved in anaerobic ATP production from phosphocreatine (CK), NADPH production (ME) and anaerobic metabolism (LDH) did not differ between active and estivating animals (Figures 2D–F).

**Figure 3:**
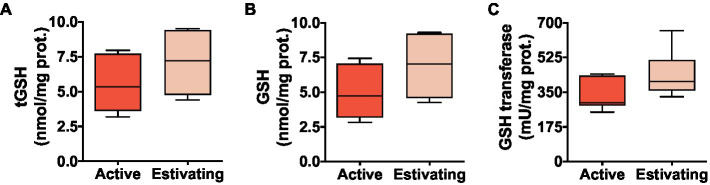
Levels of glutathione (total and reduced) and glutathione transferase activity in the skeletal muscle of *Pleurodema diplolister* collected during the dry season (estivating) or the rainy season (active). **(A)** Total glutathione (tGSH). **(B)** Reduced glutathione (GSH). **(C)** Glutathione transferase. There were no significant differences between active and estivating groups. *N*=6.

**Figure 4:**
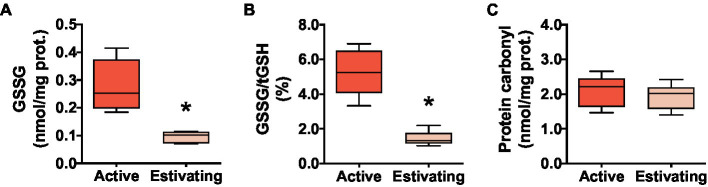
Redox imbalance and oxidative stress markers in the skeletal muscle of *Pleurodema diplolister* collected during the dry season (estivating) or the rainy season (active). **(A)** Disulfide glutathione (GSSG). **(B)** Ratio between GSSG and total glutathione (GSSG/tGSH). **(C)** Protein carbonyl. The asterisk (*) denotes significant differences between active and estivating groups. *N*=6.

There was no difference between active and estivating animals in terms of reduced glutathione (GSH) and total glutathione (tGSH=GSH+2×GSSG) concentration in skeletal muscle (Figures 3A and B). Similarly, GST, an enzyme that uses GSH to detoxify electrophilic molecules, was unaffected by estivation in *P. diplolister* (Figure 3C). Estivating *P. diplolister* had 65.5% less disulfide glutathione (GSSG) than active *P. diplolister*, which resulted in a 72.4% decrease in GSSG/tGSH ratio in the muscle of estivating animals compared with that of active animals (Figures 4A and B). The degree of oxidative damage to muscular proteins assessed as protein carbonyl levels was unaffected by estivation in *P. diplolister* (Figure 4C).

**Figure 5:**
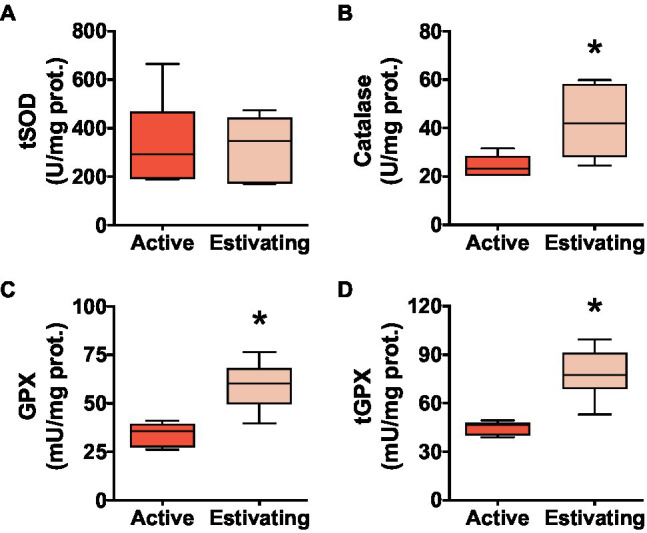
Activities of core antioxidant enzymes in the skeletal muscle of *Pleurodema diplolister* collected during the dry season (estivating) or the rainy season (active). **(A)** Total superoxide dismutase (tSOD). **(B)** Catalase. **(C)** H_2_O_2_-Glutathione peroxidase (GPX). **(D)** Total glutathione peroxidase (tGPX). The asterisk (*) denotes significant differences between active and estivating groups. *N*=6.

Total superoxide dismutase (tSOD) activity did not differ between estivating and active animals (Figure 5A). Estivating *P. diplolister* had higher activity of peroxide-detoxifying enzymes than that in the muscle of active *P. diplolister*. The activities of catalase, GPX, and tGPX were 74.4, 72.7, and 74.1% higher, respectively, in the muscle of estivating animals than in those of active animals (Figures 5B–D).

### Effect of Species and Metabolic State on Biochemical Biomarkers

To assess the effect of species (*P. diplolister* or *P. cristiceps*) and metabolic state (active or estivating) on the biochemical biomarkers measured in the skeletal muscle of active and estivating *P. diplolister* (present study) and active and estivating *P. cristiceps* collected in the same site (Moreira et al., 2020), we assembled the data (Table 1) and conducted a two-way ANOVA (Table 2). Simple main effects analyses revealed that species had an effect on the activities of CS, ICDH, ME, CK, GST, catalase, GPX and tGPX, as well as on GSSG/tGSH ratio (Table 2). In the case of metabolic state, simple main effects analyses showed that estivation had an effect on the activities of CS, ICDH, catalase, GPX and tGPX, as well as on the levels of tGSH, GSH, GSSG, and GSSG/tGSH (Table 2). Moreover, there was an interaction effect between species and metabolic state on GSSG and GSSG/tGSH levels (Table 2).

**Table 1:** Biochemical variables measured in the skeletal muscle of free-ranging *Pleurodema diplolister* and *Proceratophrys cristiceps* collected during the rainy (active) and the dry season (estivating) in the Caatinga.

Biochemical variable	*Pleurodema diplolister*		*Proceratophrys cristiceps* ^*^	
	Active	Estivating	Active	Estivating
*Metabolic enzymes*				
CS (mU/mg prot.)	553.05±178.57 (6)	355.27±123.50 (6)	313.92±73.39 (6)	199.65±56.91 (6)
ICDH (mU/mg prot.)	69.75±28.48 (6)	36.31±13.49 (6)	29.14±4.92 (6)	21.77±5.45 (6)
ME (mU/mg prot.)	8.89±2.04 (6)	9.36±1.23 (6)	11.63±3.82 (6)	12.31±3.53 (6)
PK (U/mg prot.)	4.16±0.54 (6)	4.33±0.42 (6)	4.36±0.24 (6)	3.65±0.74 (6)
LDH (U/mg prot.)	5.98±0.74 (6)	5.90±0.48 (6)	5.02±0.64 (6)	5.64±1.40 (6)
CK (U/mg prot.)	29.15±6.20 (6)	28.40±3.52 (6)	35.15±2.52 (6)	35.52±5.43 (6)
*Antioxidants*				
tGSH (nmol/mg prot.)	5.54±2.00 (6)	7.11±2.52 (6)	6.38±1.14 (6)	10.01±3.16 (6)
GSH (nmol/mg prot.)	4.99±1.89 (6)	6.92±2.49 (6)	6.02±1.12 (6)	9.71±3.04 (6)
GST (mU/mg prot.)	334.73±80.59 (6)	438.25±119.66 (6)	36.93±12.51 (6)	38.33±6.39 (6)
tSOD (U/mg prot.)	338.33±180.54 (6)	323.95±131.15 (6)	235.97±95.56 (6)	298.30±136.18 (6)
Catalase (U/mg prot.)	24.38±4.59 (6)	42.52±15.03 (6)	10.74±2.37 (5)	15.92±3.58 (6)
GPX (mU/mg prot.)	34.24±6.05 (6)	59.13±12.47 (6)	22.12±5.01 (6)	39.38±11.39 (6)
tGPX (mU/mg prot.)	45.01±4.24 (6)	78.35±15.60 (6)	30.53±6.66 (6)	47.82±12.87 (6)
*Oxidative stress markers*				
GSSG (nmol/mg prot.)	0.28±0.09 (6)	0.10±0.02 (6)	0.18±0.04 (6)	0.15±0.06 (6)
GSSG/tGSH (%)	5.23±1.35 (6)	1.44±0.42 (6)	2.83±0.70 (6)	1.51±0.21 (6)
Carbonyl (nmol/mg prot.)	2.11±0.44 (6)	1.94±0.36 (6)	2.13±0.67 (6)	2.30±0.54 (6)

* *Biochemical data for active and estivating Proceratophrys cristiceps are from Moreira et al. (2020). Data are shown as mean±standard deviation (N). CS, citrate synthase; ICDH, isocitric dehydrogenase (NADPH); ME, malic enzyme; PK, pyruvate kinase; LDH, lactic dehydrogenase; CK, creatine kinase; tGSH, total glutathione; GSH, reduced glutathione; GST, glutathione transferase; tSOD, total superoxide dismutase; GPX, glutathione peroxidase (H_2_O_2_); tGPX, total glutathione peroxidase; GSSG, disulfide glutathione; carbonyl, protein carbonyl*.

**Table 2:** Two-way ANOVA of the biochemical variables measured in the skeletal muscle of free-ranging *Pleurodema diplolister* and *Proceratophrys cristiceps* collected during the rainy (active) and the dry season (estivating) in the Caatinga.

Biochemical variable	Two-way ANOVA results					
	Species		Metabolic state		Interaction	
	*F*	*p*	*F*	*p*	*F*	*p*
*Metabolic enzymes*						
CS	16.77	**0.0006**	10.48	**0.0041**	0.75	0.3966
ICDH	17.42	**0.0005**	9.54	**0.0058**	3.90	0.0624
ME	5.95	**0.0242**	0.24	0.6286	0.01	0.9299
PK	1.33	0.2617	1.63	0.2162	4.30	0.0513
LDH	2.85	0.1067	0.548	0.4678	0.91	0.3514
CK	11.91	**0.0025**	0.01	0.9210	0.09	0.7715
*Antioxidants*						
tGSH	3.88	0.0629	7.51	**0.0126**	1.19	0.2892
GSH	4.33	0.0506	9.33	**0.0063**	0.91	0.3519
GST	139.00	**<0.0001**	3.14	0.0915	2.98	0.0998
tSOD	1.27	0.2733	0.18	0.6775	0.46	0.5073
Catalase	33.24	**<0.0001**	11.17	**0.0034**	3.45	0.0788
GPX	17.57	**0.0005**	30.75	**<0.0001**	1.01	0.3278
tGPX	25.79	**<0.0001**	32.64	**<0.0001**	3.29	0.0849
*Oxidative stress markers*						
GSSG	0.78	0.3873	18.00	**0.0004**	10.37	**0.0043**
GSSG/tGSH	12.96	**0.0018**	61.99	**<0.0001**	14.44	**0.0011**
Protein carbonyl	0.86	0.3655	<0.01	0.9969	0.63	0.4377

*Metabolic state=active or estivating. Species=Pleurodema diplolister or Proceratophrys cristiceps. Interaction=metabolic state×species. Biochemical data for active and estivating Proceratophrys cristiceps were obtained from Moreira et al., 2020. CS, citrate synthase; ICDH, isocitric dehydrogenase (NADPH); ME, malic enzyme; PK, pyruvate kinase; LDH, lactic dehydrogenase; CK, creatine kinase; tGSH, total glutathione; GSH, reduced glutathione; GST, glutathione transferase; tSOD, total superoxide dismutase; GPX, glutathione peroxidase (H_2_O_2_); tGPX, total glutathione peroxidase; GSSG, disulfide glutathione; carbonyl, protein carbonyl. Significant effects (p<0.05) are highlighted in bold*.

### Principal Component Analysis and Correlations

**Figure 6:**
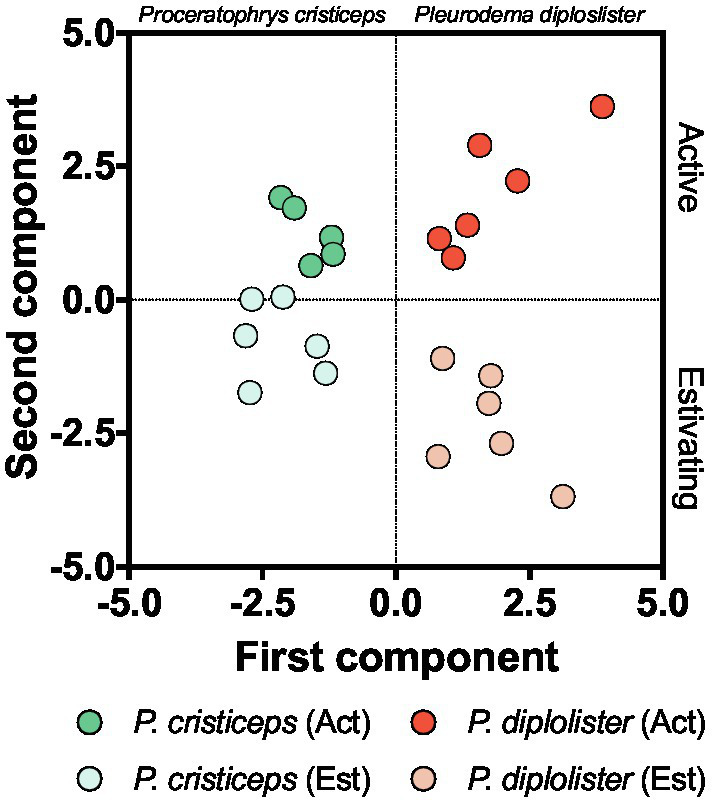
Principal component analysis of biomarkers of key metabolic pathways, endogenous antioxidants, redox imbalance, and oxidative stress. Each circle corresponds to an individual. Note that the group of active *Proceratophrys cristiceps* has only five individuals, since one of the analyzed variables (catalase activity) was measured in only five animals in this group. The resulting coefficients for each principal component are shown in Table 3. Data for *P. cristiceps*, previously published in Moreira et al. (2020) and summarized in Table 1, were included in the analysis.

The PCA with all biochemical variables, except for tGSH levels, identified two major components that effectively distinguished individual animals in terms of metabolic state (activity vs. estivation) and species (*P. diplolister* vs. *P. cristiceps*; Figure 6). Active *P. diplolister* clustered in the upper right quadrant, estivating *P. diplolister* in the lower right, active *P. cristiceps* in the upper left and estivating *P. cristiceps* in the lower left. The first component, which separated the species, was mainly a contribution of CS, ICDH, and GST activities (Table 3). On the other hand, the second component, which separated active animals from estivating animals regardless of the species, was mainly a contribution of GSSG/tGSH ratio, GPX activity, and tGPX activity (Table 3).

**Table 3:** Principal components and coefficients for each biochemical variable measured in the skeletal muscle of active and estivating frogs of the two species.

Biochemical variable	Component				
	PC1	PC2	PC3	PC4	PC5
*Metabolic enzymes*					
Citrate synthase	**0.364**	0.263	0.164	−0.040	0.061
Isocitric dehydrogenase	**0.358**	0.281	0.136	−0.060	0.014
Malic enzyme	−0.212	−0.063	**0.447**	0.261	0.248
Pyruvate kinase	0.153	0.058	**−0.394**	**0.476**	0.008
Lactic dehydrogenase	0.137	−0.064	0.155	**0.638**	0.150
Creatine kinase	−0.329	0.010	−0.285	−0.282	−0.026
*Endogenous antioxidants*					
Reduced glutathione^*^	−0.247	−0.237	0.150	**0.325**	**−0.383**
Glutathione transferase	**0.447**	−0.136	−0.046	−0.008	−0.078
Superoxide dismutase	0.094	−0.043	**0.610**	−0.223	0.150
Catalase	0.333	−0.307	−0.104	−0.003	−0.094
Glutathione peroxidase (H_2_O_2_)	0.215	**−0.413**	0.031	−0.138	−0.197
Glutathione peroxidase	0.247	**−0.415**	−0.011	−0.089	−0.162
*Oxidative stress markers*					
Disulfide glutathione	0.035	0.358	0.000	0.164	**−0.443**
GSSG/tGSH	0.201	**0.441**	−0.010	−0.060	−0.152
Protein carbonyl	−0.121	0.063	0.292	−0.016	**−0.666**

*The top-3 coefficients for each component are highlighted in bold*.

* *Considering the strong (R^2^=0.9960) and significant (Pearson correlation, p<0.0001) correlation between total glutathione (tGSH) and reduced glutathione (GSH), we excluded tGSH levels and used only GSH levels in the PCA*.

## Discussion

Several amphibians rely on metabolic depression to spare resources and survive in the “too hot and too dry” conditions of the Brazilian Caatinga. Here, we confirmed the suppression of oxidative metabolism pathways during estivation and found that estivating *P. diplolister* frogs collected during the dry season have increased activity of key antioxidant enzymes compared with those of frogs collected during the rainy season. These observations indicate that, similar to *P. cristiceps*, *P. diplolister* also present the POS mechanism during estivation. In fact, when data for the two species were analyzed together, we found an effect of estivation on several endogenous antioxidants, whose levels were higher in the muscle estivating frogs than those in active frogs. While no changes in oxidative damage to proteins were observed, estivating *P. diplolister* had lower levels of GSSG and GSSG/tGSH in the skeletal muscle than those observed in active individuals of the same species. From the assembly and analysis of the present results for *P. diplolister* with those previously published for another Caatinga species (*P. cristiceps*), several biochemical peculiarities emerged. Many biomarkers, including metabolic and antioxidant enzymes, varied between species. Such discrepancies might underlie the physiological and behavioral differences between these two species that share the same microhabitat and survival strategy (i.e., to estivate) during the dry season.

### Metabolic Adaptations

The decrease in the activity of two enzymes associated with oxidative metabolism (CS and ICDH) in the muscle of both *P. diplolister* and *P. cristiceps* (Moreira et al., 2020) is in line with the metabolic adaptations of estivation. The suppression of oxygen-dependent metabolic pathways and whole-animal oxygen consumption are hallmarks of estivation (Guppy and Withers, 1999). Indeed, resting metabolic rate of both *P. diplolister* and *P. cristicep*s decrease by ~50% in the dry season (i.e., during estivation) when compared with that measured during the rainy season (Pereira, 2016). In Australian frogs *Cyclorana alboguttata*, oxygen consumption by either the whole animal or saponin-permeabilized skeletal muscle fibers is strongly suppressed after 4 months of estivation (Reilly et al., 2014). Changes in CS activity during the estivation of *C. alboguttata* depends on specific muscle functions (i.e., in which specific muscle the measurement is made). For example, the activity of CS decreases in sartorius, iliofibularis, and rectus abdominus muscles, whereas it increases in gastrocnemius, of *C. alboguttata* estivating for 6months when compared with control animals (Mantle et al., 2010). Estivation is also part of the life history of lungfish, when whole-organism oxygen consumption declines (Fishman et al., 1992). Accordingly, muscle CS activity decreases as the African lungfish *Protopterus dolloi* enters the hypometabolic phase of estivation (Frick et al., 2008b). Overall, our findings with anurans collected directly in the field corroborate previous observations of decreased oxidative metabolism in other vertebrates induced to estivate in laboratory conditions.

With a reduction in oxidative metabolism, one might expect compensatory adjustments of oxygen-independent alternative pathways. Here, we found no changes in the activities of PK, CK, and LDH in the muscle of *P. diplolister* and no effect of metabolic state (i.e., estivation) on these enzymes. In *P. cristiceps*, a very similar response was observed, when muscular CK and LDH activities remained unchanged, whereas PK activity had a minor decrease, in estivating animals compared with the control group (Moreira et al., 2020). The responses of these enzymes in Caatinga frogs agree with previous observations in *C. alboguttata*, where, depending on muscle type, LDH activity is similar or lower in estivating frogs compared with that in the active group (Mantle et al., 2010). In the African lungfish, the capacity of the glycolytic pathway, measured as PK and hexokinase activities, is also maintained in skeletal muscle during estivation (Frick et al., 2008a). Moreover, LDH activity in the skeletal muscle of *P. dolloi* is not affected by estivation (Frick et al., 2008a). These findings indicate that, despite reduced oxygen consumption, the downregulation of ATP-expensive pathways maintains energetic supply/demand balance and oxygen availability is not a significant constraint during estivation for these animals. Indeed, the oxygen concentration inside the burrows of estivating frogs in the Caatinga does not fall below 20.7% down to 1.5m depth (Carvalho et al., 2010). However, the biochemical responses of these metabolic pathways differ in the North American spadefoot toad, whose estivation is associated with large increases in PK and CK activities in skeletal muscle (Cowan et al., 2000). Possible muscle functionality differences aside, *S. couchii* seems to differ from *C. alboguttata*, *P. dolloi*, and the Caatinga frogs in terms of biochemical adaptations associated with energy metabolism during estivation. Once again, our findings from field-collected animals corroborate previous observations of the lack of compensatory upregulation of oxygen-independent pathways in other vertebrates induced to estivate under laboratory conditions.

### Redox Adaptations

Glutathione is a cofactor for antioxidant enzymes and an antioxidant itself. This tripeptide is present at millimolar intracellular concentrations in animal tissues, where it can be found in its reduced (GSH) and disulfide (GSSG) free forms. More important than their absolute levels, the ratio GSSG/tGSH is a reliable indicator of disturbances in redox balance (Flohé, 2013). For both *P. cristiceps* (Moreira et al., 2020) and *P. diplolister*, the skeletal muscle had a more reduced cellular environment months after the onset estivation (i.e., lower GSSG/tGSH values) compared with that of animals assessed during the active season. Muscular GSSG/tGSH ratio behaves differently in other estivating vertebrates. The spadefoot toad *S. couchii* experience no changes in GSSG/tGSH levels after 2months of estivation (Grundy and Storey, 1998). In the muscle of another African lungfish, *Protopterus annectens*, GSSG/tGSH increases sharply after 6months under estivation (Ong, 2016). The decrease in GSSG/tGSH observed in frogs estivating in the Caatinga can be explained by the expected decrease in the production of reactive oxygen species once the hypometabolic state is achieved and oxygen consumption stabilize at a slow rate (i.e., a slow electron flow through the mitochondrial electron transport chain). Indeed, the skeletal muscle of estivating *C. alboguttata* frogs produces less hydrogen peroxide when compared with active frogs (Reilly et al., 2014). The behavior of GSSG/tGSH in the muscle of freely estivating Caatinga frogs is consistent with a more reduced intracellular redox state that should favor muscle preservation during estivation, as observed for *C. alboguttata* (Hudson and Franklin, 2002; Young et al., 2013; Reilly et al., 2014).

In addition to GSSG/tGSH, we also assessed levels of oxidative damage to proteins in the muscle of Caatinga frogs. For both *P. cristiceps* (Moreira et al., 2020) and *P. diplolister*, protein carbonyl levels remained stable months after the onset of estivation. Protein carbonyl and malondialdehyde (a lipid peroxidation marker) levels are maintained in the gastrocnemius muscle of *C. alboguttata* frogs during estivation (Young et al., 2013). Likewise, the degree of oxidative damage to lipids in skeletal muscle (assessed as TBARS and hydroperoxide levels) does not differ between freshwater controls and 6-month estivating *P. annectens* (Ong, 2016). Protein carbonyl levels decrease by 45% in the muscle of *P. annectens* after 6months of estivation (Ong, 2016). On the other hand, the levels of two biomarkers of oxidative damage to lipids increase in the muscle of *S. couchii* estivating for 2months compared with its active control group (Grundy and Storey, 1998) and protein carbonyl content increases in the iliofibularis muscle, but not in gastrocnemius, from estivating frogs compared with control frogs (Young et al., 2013). Our findings indicate the lack of accumulation of oxidative damage to biomolecules in the skeletal muscle of freely estivating Caatinga frogs despite the monthslong estivation period spent burrowed.

Regardless of the reduced intracellular redox state and the lack of oxidative damage (at the time the animals were collected), both *P. cristiceps* (Moreira et al., 2020) and *P. diplolister* had higher activities of peroxide-detoxifying enzymes during estivation than those during activity in the rainy season. In fact, the second principal component of PCA, which separated active from estivating individuals of both species, was mainly a contribution of three variables directly related to redox metabolism: tGPX, GPX, and GSSG/tGSH. Moreover, when data from both species were analyzed together, we detected an effect of metabolic state (i.e., estivation) on catalase, GPX and tGPX activities. The modulation of antioxidant enzymes during estivation has been studied in the skeletal muscle of a few vertebrate species. Similar to Caatinga frogs, GPX and tGPX activities increase and tSOD activity is maintained after 6months of estivation in the muscle of *P. annectens* compared with the control group (Ong, 2016). In the case of *S. couchii*, toads estivating for 2months have increased in SOD and tGPX activities in skeletal muscle compared with those of active toads; other four antioxidants had their activities maintained at control levels (Grundy and Storey, 1998). For the Australian burrowing frog, total antioxidant capacity of both water-soluble and membrane-bound fractions of skeletal muscle does not differ between active and 6-month-estivating frogs (Hudson et al., 2006). At the transcript level, the number of transcripts for catalase and glutathione peroxidase remains stable, whereas that for mitochondrial superoxide dismutase decrease, in skeletal muscle of estivating *C. alboguttata* compared with the levels measured in control frogs (Hudson et al., 2006). The activity of both mitochondrial and cytosolic SOD, however, is higher in the muscle of estivating frogs than in that of control frogs (Young et al., 2013). The antioxidant boost in the muscle of estivating frogs is further corroborated by the observation of the upregulation of several genes under the regulation of Nrf2 (Reilly et al., 2013), a transcription factor that controls the expression of antioxidants and other cytoprotective genes (Kaspar et al., 2009; Niture et al., 2014). The measurement of antioxidant levels in freely estivating *P. diplolister* confirmed the activation of the endogenous antioxidant machinery during estivation as observed in other vertebrate models of estivation.

### Preparation for Oxidative Stress

The upregulation of endogenous antioxidants is a hallmark of the biochemical responses that allow animals to adapt and survive adverse environmental conditions. This biochemical phenomenon was first observed in snakes exposed to freezing or anoxia, and later confirmed in a few more species of vertebrates and invertebrates, being coined in 1998 as POS (Hermes-Lima and Storey, 1993; Hermes-Lima et al., 1998). Since then, the occurrence of POS in animals exposed to adverse ambient has been shown to be widespread in more than 80 animal species distributed in eight phyla (Hermes-Lima and Zenteno-Savín, 2002; Welker et al., 2013; Moreira et al., 2016, 2017). Despite recent advances in the field, studies reporting the POS phenotype have been mostly performed under artificial laboratory settings (Hermes-Lima et al., 2015; Giraud-Billoud et al., 2019) and only recently studies on the modulation of antioxidants in animals under ecophysiological relevant natural scenarios have been published. For example, mussels *Brachidontes solisianus* stimulate glutathione synthesis after 4h of air exposure (a condition that impairs oxygen uptake) in a rocky beach under natural settings (Moreira et al., 2021). In the case of *Astyanax elachylepis*, fish collected in a naturally hypoxic intermittent stream suffer more oxidative damage and have increased GPX activity in gills compared with fish living in a naturally normoxic perennial stream during the dry season (Ondei et al., 2020). More recently, the levels of redox metabolism biomarkers in naturally hibernating common Asian toads, *Duttaphrynus melanostictus*, at the end of the winter were compared with those in naturally active toads collected during summer (Patnaik and Sahoo, 2021). The redox balance in the brain and liver of hibernating toads shifts toward a more oxidized state (i.e., increased GSSG/GSH ratio; Patnaik and Sahoo, 2021). Such a disruption of the redox balance is associated with increased damage to proteins and lipids and the induction of catalase activity in both tissues of hibernating toads compared with active toads (Patnaik and Sahoo, 2021). Therefore, evidence from field-collected mussels, fish and anurans, including our previous (Moreira et al., 2020) and present study, indicates the occurrence of POS under ecologically relevant settings.

We have previously reported the upregulation of selected antioxidants in the muscle of *P. cristiceps* estivating under natural conditions (Moreira et al., 2020). Here, we measured the same biochemical variables in the muscle of another anuran species that share the same microhabitat in the Brazilian Caatinga during the dry seasons when they both estivate. Most biochemical changes observed in *P. diplolister* matched those observed in *P. cristiceps*: downregulation of CS, ICDH, and GSSG/tGSH levels; upregulation of catalase, GPX and tGPX activities; and the maintenance of ME, LDH, CK, GST, protein carbonyl, and tSOD levels in estivating vs. active frogs. Responses observed exclusively in *P. cristiceps* were the small decrease in PK activity and the increase in GSH concentration in estivating vs. active frogs (Moreira et al., 2020). Although GSSG/tGSH decreased in both Caatinga species, the underlying causes differed between *P. diplolister* and *P. cristiceps*. While in *P. diplolister* the reduction in GSSG/tGSH was driven by a decrease in GSSG concentration, in *P. cristiceps* the cause was an increase in GSH levels in estivating vs. active frogs (Moreira et al., 2020). This indicates that, despite the resource restriction in food and water in the Caatinga, *P. cristiceps* stimulate GSH synthesis during estivation, a response that is not shared with *P. diplolister*. Moreover, given the significant interaction (species×metabolic state) effects on GSSG and GSSG/tGSH, it appears that estivation affects glutathione redox balance in a species-specific manner.

### Comparison Between the Two Anuran Species: Ecology and Physiology

In addition to the few different biochemical responses associated with the estivation phenotype, we also found significant effects of species on some biochemical variables. When comparing species, the skeletal muscle of *P. diplolister* appeared to have higher levels of aerobic metabolic enzymes than that of *P. cristiceps*. Accordingly, the first principal component of the PCA, which separated species into two distinct groups, was mainly a contribution of CS, ICDH, GST activities. These findings agree with the discrepancy in whole-animal oxygen consumption between *P. diplolister* (~0.25mlg^−1^ h^−1^) and *P. cristiceps* (~0.065mlg^−1^ h^−1^) collected during activity in the rainy season (Pereira, 2016). Noteworthy, estivating *P. diplolister* immediately resumes activity when disturbed, whereas estivating *P. cristiceps* takes minutes to show any sign of activity. Such a “more active/oxidative” characteristic of *P. diplolister* is associated with a more oxidized intracellular redox state in skeletal muscle (i.e., higher GSSG/tGSH ratio) and higher levels of antioxidant enzymes than those of *P. cristiceps*.

The ability to dig out the soil, associated with muscle conservation, is a vital point at the end of the estivation of the amphibians that are buried (Grundy and Storey, 1998). In this sense, there are metabolic differences associated with energy production and redox balance between *P. diplolister* and *P. cristiceps* in relation to the conservation of muscle tone. Although there is a significant decrease in the capacity of oxidative pathways in muscle in both species, *P. diplolister* tends to maintain higher levels of both anaerobic and aerobic metabolism than *P. cristiceps* does. This evidence may be relevant within the ecological relationships between these species because buried individuals of *P. diplolister* can react quickly to environmental signals of the first rains at the end of the dry season, having quick access to the reproduction areas which will not be used simultaneously by *P. cristiceps*, which represents an example of niche segregation by space (dos Santos Protázio et al., 2015; Jared et al., 2019; Lima et al., 2019).

The genus *Pleurodema* is endemic to South America, with several representatives in environments with low water availability and contrasting thermal extremes, such as *P. brachyops* in xerophytic forests in Colombia or *P. bufoninum* in the cold Argentine steppes (Faivovich et al., 2012). Within this context, the mechanisms associated with estivation in *P. diplolister* could be more associated with a phylogenetic trait than with a convergent evolution with the environmental conditions of the caatingas. Moreover, the high activity of metabolic enzymes in the skeletal muscle of *P. diplolister* (compared with those in *P. cristiceps*) can also be related to the rapid onset of reproductive activity on the arrival of the rainy season. Although the first rains form incipient water bodies, these are sufficient to allow the reproduction of *P. diplolister* (Hödl, 1992; Jared et al., 2019). Unlike this, *P. cristiceps* only comes out of estivation in the presence of heavy rain, since adults need greater availability of aquatic microhabitats for their reproduction and the deposition of eggs occurs in a place and vocalization in another (Jared et al., 2019). The results found for *P. diplolister* and *P. cristiceps* during estivation may be an example of how the richness of physiological mechanisms enhances the overlap of niches during the dry season, while during times of activity there are possibly ecological processes of competition.

## Conclusion

**Figure 7:**
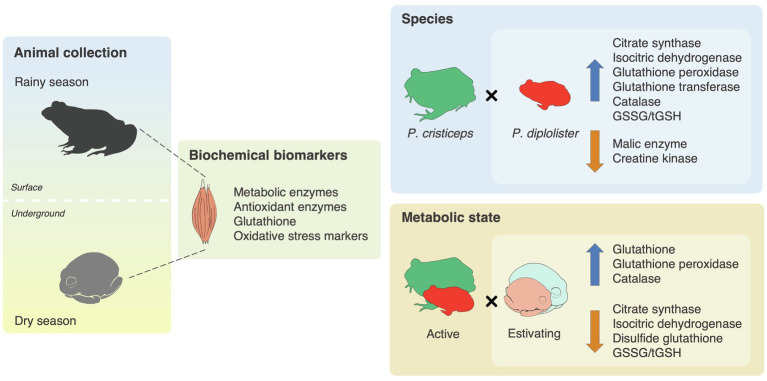
Graphical summary of the biochemical adaptations of two frog species that estivate during the seasonal droughts in the Caatinga. Free-ranging adult specimens of *Pleurodema diplolister* and *Proceratophrys cristiceps* were collected during the rainy (active) and the dry season (estivating) in the Caatinga. The whole skeletal mass surrounding the femur was dissected and homogenized for the measurement of biochemical variables. The activities of intermediary metabolism and antioxidant enzymes, the levels of total (tGSH), reduced (GSH) and disulfide (GSSG) glutathione, and the degree of oxidative damage to proteins were measured. Species had an effect on the activities of citrate synthase, isocitric dehydrogenase, glutathione peroxidase, glutathione transferase, catalase, malic enzyme and creatine kinase, as well as on GSSG/tGSH ratio. Estivation had an effect on the activities of citrate synthase, isocitric dehydrogenase, glutathione peroxidase and catalase, as well as on the levels of tGSH, GSH, GSSG, and GSSG/tGSH. The upward arrows indicate variables whose values are higher in *P. diplolister* (vs. *P. cristiceps*) or in estivating frogs (vs. active frogs). The downward arrows indicate variables whose values are lower in *P. diplolister* (vs. *P. cristiceps*) or in estivating frogs (vs. active frogs).

We have shown that *P. diplolister* enhances its muscular antioxidants during estivation under field conditions, being another species in the list of animals that prepare for oxidative stress during estivation. We also found that two phylogenetically distant anuran species that share the same microhabitat and take advantage of entering estivation to endure the dry season in the Brazilian Caatinga have similar biochemical responses during metabolic depression (Figure 7). Still, the two species diverge in many biochemical indicators, a possible correlate of their contrasting behavior and physiology. The Caatinga frogs seem to balance the suppression of oxidative pathways (to reduce oxygen demand), the maintenance of the capacity of oxygen-independent pathways and the activation of cytoprotective pathways (i.e., antioxidants) to preserve muscle function and explore the windows of opportunity created by the unpredictable arrival of heavy rains.

## Data Availability Statement

The raw data supporting the conclusions of this article will be made available by the authors, without undue reservation.

## Ethics Statement

The animal study was reviewed and approved by Animal Care and Use Committee, Universidade de São Paulo, São Paulo, Brazil.

## Author Contributions

DM: data curation, formal analysis, investigation, visualization, writing – original draft, and writing – review and editing. JC-F: formal analysis, investigation, writing – original draft, and writing – review and editing. CN: conceptualization, funding acquisition, resources, and writing – review and editing. JC: conceptualization, resources, and writing – review and editing. MH-L: conceptualization, funding acquisition, project administration, resources, visualization, and writing – review and editing. All authors contributed to the article and approved the submitted version.

## Funding

This work was supported by grants from Fundação de Amparo à Pesquisa do Estado de São Paulo (FAPESP, Brazil, grant 2014/16320-7 to CN and 2020/12962-5 to JC), Instituto Nacional de Ciência e Tecnologia – Fisiologia Comparada (Brazil, grant 08/57712-4 to JC), Fundação de Apoio à Pesquisa do Distrito Federal (FAPDF, Brazil, grant 193.00000219/2019-71 to MH-L), and Conselho Nacional de Desenvolvimento Científico e Tecnológico (CNPq, Brazil, grant 421384/2018-2 to MH-L). This study was financed in part by Coordenação de Aperfeiçoamento de Pessoal de Nível Superior (CAPES, Brazil, finance code 001 to DM and the PNPD/CAPES Scholarship 23106.055368/2017-89 to JC-F).

## Conflict of Interest

The authors declare that the research was conducted in the absence of any commercial or financial relationships that could be construed as a potential conflict of interest.

## Publisher’s Note

All claims expressed in this article are solely those of the authors and do not necessarily represent those of their affiliated organizations, or those of the publisher, the editors and the reviewers. Any product that may be evaluated in this article, or claim that may be made by its manufacturer, is not guaranteed or endorsed by the publisher.
